# Isolated Oral and Esophageal Mucosal Injury as a Rare Oxcarbazepine Adverse Drug Reaction: A Case Report

**DOI:** 10.7759/cureus.117018

**Published:** 2026-09-27

**Authors:** Joselyn De Jesus-Gonzalez, Quinten P Federico, Patrick Malafronte, Anne Champeaux, Shreya Narayanan, John Jacobs

**Affiliations:** 1 Department of Internal Medicine, Morsani College of Medicine, University of South Florida, Tampa, USA; 2 Department of Pathology and Laboratory Medicine, Tampa General Hospital, Tampa, USA; 3 Department of Pathology and Cell Biology, Morsani College of Medicine, University of South Florida, Tampa, USA; 4 Division of Digestive Disease and Nutrition, Morsani College of Medicine, University of South Florida, Tampa, USA; 5 Division of Digestive Diseases and Nutrition, Joy McCann Culverhouse Center for Swallowing Disorders, Morsani College of Medicine, University of South Florida, Tampa, USA

**Keywords:** dysphagia, medication induced hypersensitivity reaction, oral mucosal injury, oxcarbazepine adverse drug reaction, severe esophagitis

## Abstract

Drug-induced mucosal injury may result from direct contact toxicity or immune-mediated adverse drug reactions. Although oxcarbazepine is a recognized etiology of severe hypersensitivity reactions, isolated oroesophageal mucosal injury without cutaneous or systemic involvement has not been previously described. We report a 68-year-old woman who developed severe diffuse esophagitis with concomitant oral mucosal ulceration approximately two weeks after oxcarbazepine initiation, with symptom onset within 24 hours of dose escalation from 150 to 300 mg twice daily. Histologic evaluation demonstrated predominantly lymphocytic esophageal inflammation with no evidence of infectious, eosinophilic, or immunobullous disease. The patient had no rash, peripheral eosinophilia, or systemic organ involvement. A Naranjo score of 7 supported a probable adverse drug reaction. Treatment consisted of discontinuation of oxcarbazepine alone, without the need for immunosuppressive or additional pharmacologic therapy. At five-month follow-up, the patient achieved endoscopic and histologic resolution. This case expands upon the recognized spectrum of oxcarbazepine-associated adverse drug reactions and emphasizes the importance of medication review in patients presenting with diffuse esophagitis and oral mucositis.

## Introduction

Acute oral mucositis with severe esophagitis is an uncommon presentation that requires consideration of infectious, inflammatory, autoimmune, dermatologic, and drug-induced etiologies. Drug-induced mucosal injury may result from direct contact toxicity or immune-mediated adverse drug reactions, the latter of which can be difficult to recognize because of their variable and often unpredictable clinical presentation [1-2]. Oxcarbazepine, an aromatic anticonvulsant, is a recognized cause of immune-mediated hypersensitivity reactions, including Stevens-Johnson syndrome (SJS), toxic epidermal necrolysis (TEN), and drug reaction with eosinophilia and systemic symptoms (DRESS) [1,3,4]. Gastrointestinal mucosal involvement in this hypersensitivity spectrum has been reported only in the context of systemic hypersensitivity syndromes with eosinophilic histology. However, isolated oroesophageal mucosal injury without cutaneous or systemic involvement has not been previously described. We report a 68-year-old woman who developed severe diffuse esophagitis with concomitant oral mucosal ulceration after oxcarbazepine exposure. Awareness of this probable adverse effect and its distinction from other causes of diffuse esophagitis is clinically relevant for gastroenterologists and internists encountering similar presentations.

## Case presentation

A 68-year-old woman with a history of paroxysmal atrial fibrillation on apixaban, hypothyroidism on levothyroxine, and recently diagnosed vestibular paroxysmia presented to an outside emergency department with oral ulcers, dysphagia, and odynophagia. She had no prior history of similar symptoms. She denied fever, systemic symptoms, recent travel, sick contacts, or caustic ingestion. Three weeks before presentation, she was started on oxcarbazepine 150 mg twice daily for vestibular paroxysmia. One week before presentation, the dose was increased to 300 mg twice daily. After this dose increase, she developed pruritus of the lips, which progressed over the following days to oral ulceration, lip edema, and an abnormal sensation of the tongue. She was evaluated at an outside dermatology clinic for suspected herpes simplex virus (HSV) infection, and empiric valacyclovir was prescribed without improvement. Given concern for SJS, she was transferred to our tertiary care center for further evaluation. On the day of transfer, oxcarbazepine was discontinued.

On arrival, she was afebrile and hemodynamically stable. Physical examination showed dry, crusted lips with mild edema and erythema of the inner labial mucosa. The oral mucosa demonstrated scattered whitish streaks and scattered ulcers involving the buccal mucosa and hard palate (Figure 1). There was no conjunctival injection, genital mucosal involvement, rash, targetoid lesions, bullae, or skin tenderness to palpation. Nikolsky sign was negative. Laboratory evaluation showed no leukocytosis or peripheral eosinophilia, and liver chemistries were within normal limits (Table 1). Dermatology was consulted and assessed a low likelihood of SJS or TEN based on the absence of cutaneous and systemic involvement.

**Figure 1:**
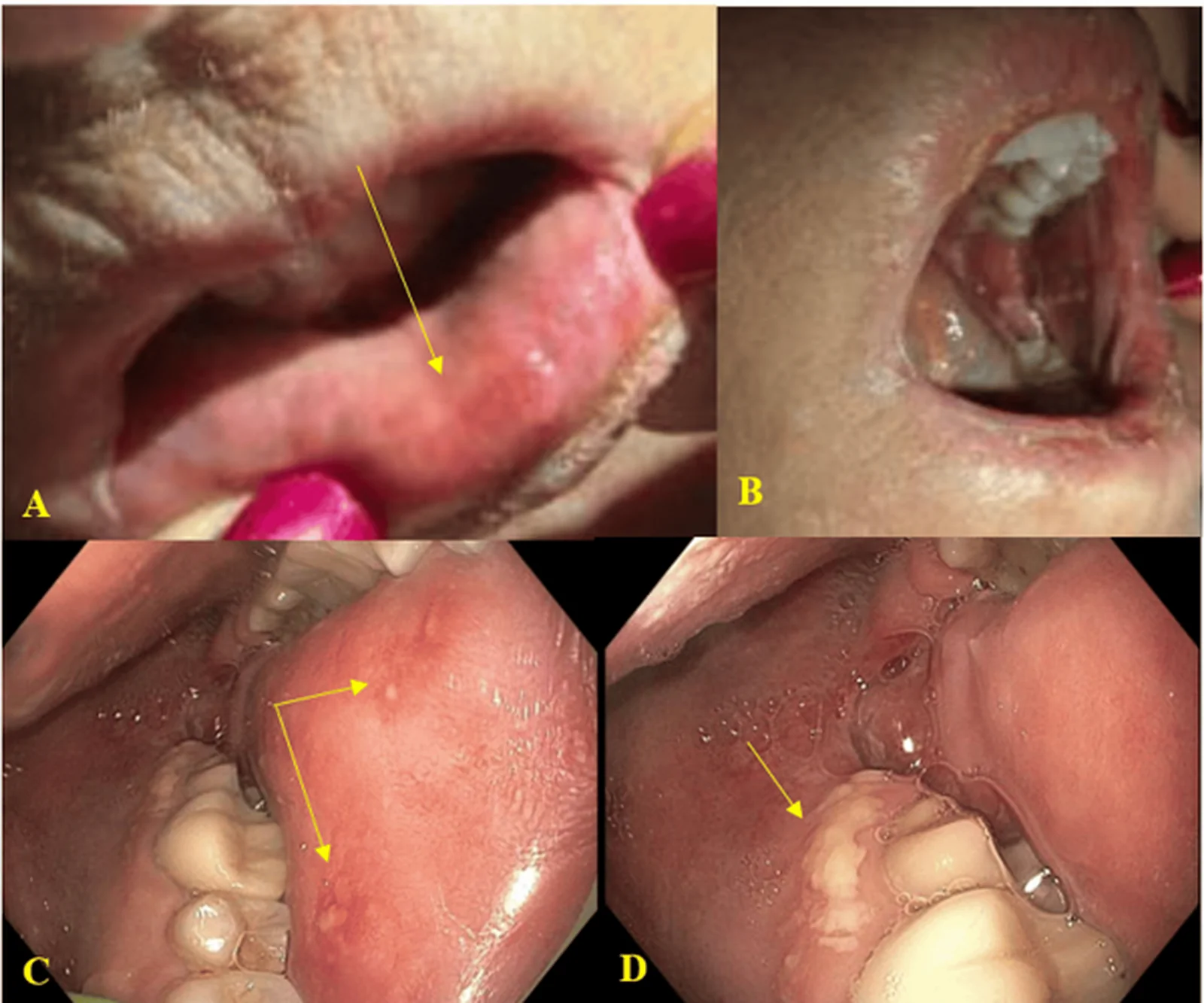
Oral mucosal involvement. Clinical images showing erythematous mucositis with superficial erosive changes involving the lower labial mucosa (A), accompanied by cheilitis with perioral crusting and fissuring (B). Scattered shallow erosions/ulcerations are seen along the buccal mucosa (C), and the hard palate demonstrates erythematous mucosal inflammation with focal whitish fibrinous slough/exudate (D).

**Table 1:** Laboratory findings.

Parameter	Result	Reference range
White blood cell count	7.23 x 10^3^/uL	4.60-10.20 x 10^3^/uL
Hemoglobin	12.1 g/dL	12.2-16.2 g/dL
Hematocrit	36.7%	37.7%-47.9%
Platelet count	231 x 10^3^/uL	142-424 x 10^3^/uL
Total neutrophils	3.88 x 10^3^/uL	1.79-7.85 x 10^3^/uL
Total lymphocytes	1.98 x 10^3^/uL	0.69-4.79 x 10^3^/uL
Total basophils	0.04 x 10^3^/uL	0.00-0.20 x 10^3^/uL
Total eosinophils	0.32 x 10^3^/uL	0.00-0.61 x 10^3^/uL
Aspartate aminotransferase	17 U/L	5-34 U/L
Alanine aminotransferase	20 U/L	0-34 U/L

Gastroenterology was consulted given the acute onset of dysphagia and odynophagia with oral mucosal involvement. Esophagogastroduodenoscopy (EGD) demonstrated diffuse esophageal mucosal erythema, congestion, luminal narrowing, tight circumferential folds, and multiple esophageal webs with progressive worsening distally (Figure 2). Histopathologic examination of esophageal biopsies demonstrated benign squamous mucosa with focal ulceration, parakeratosis, and mild lymphocytic inflammation, most prominent in the superficial epithelial layers and more pronounced in the distal esophagus (Figure 3). Grocott methenamine silver stain was negative for fungal elements, and immunohistochemical stains for HSV and cytomegalovirus (CMV) were negative. Direct immunofluorescence (DIF) performed on specimens submitted in Michel’s solution demonstrated no IgG, IgM, IgA, or C3 deposition.

**Figure 2:**
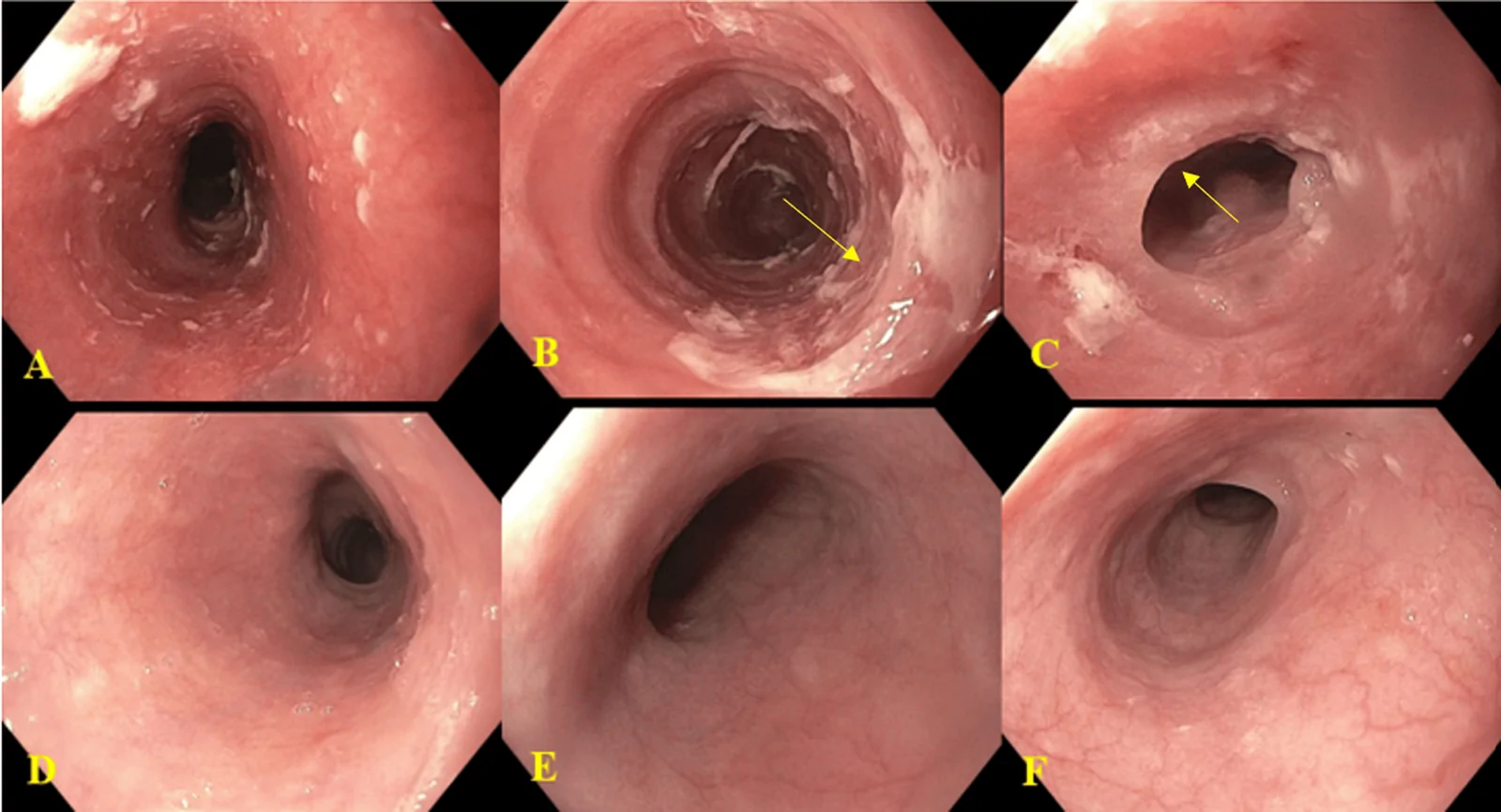
Endoscopic findings of diffuse esophageal injury and interval healing. Initial upper endoscopy showed severe diffuse esophagitis involving the upper esophagus (A), mid-esophagus (B), and lower esophagus (C). Findings included mucosal erythema, granularity, congestion, superficial sloughing (yellow arrow, B), overlapping esophageal webs (yellow arrow, C), and significant luminal narrowing (B, C), most prominent distally. Follow-up endoscopy five months after oxcarbazepine discontinuation showed marked mucosal healing, with resolution of sloughing and inflammatory narrowing and restoration of a patent lumen in the upper esophagus (D), mid-esophagus (E), and lower esophagus (F).

**Figure 3:**
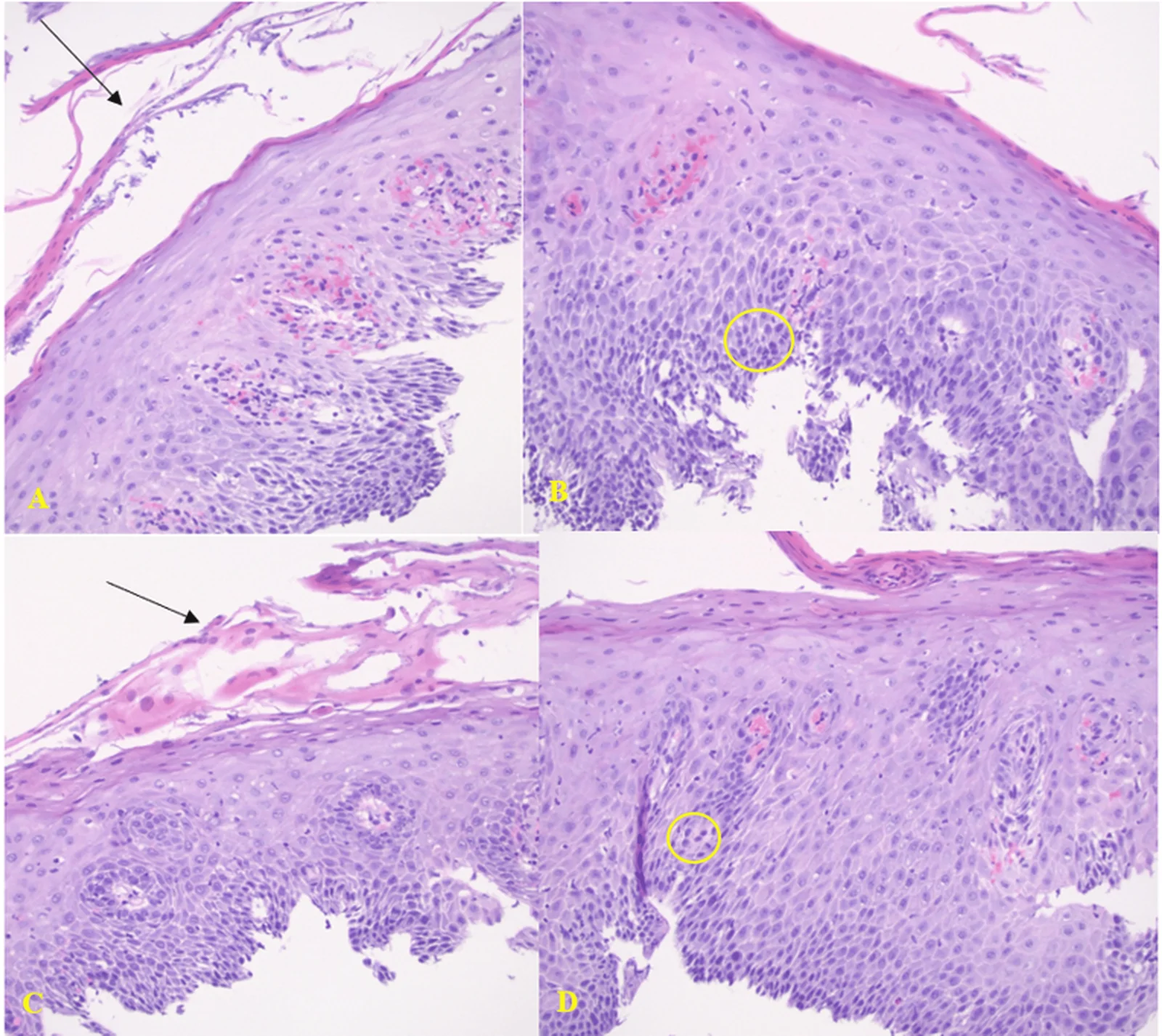
Histopathologic findings from esophageal biopsies obtained during the index upper endoscopy. Hematoxylin and eosin-stained sections obtained during the index upper endoscopy from the proximal esophagus (A, B) and distal esophagus (C, D) demonstrate benign squamous mucosa with focal superficial erosion and ulceration (black arrows), parakeratosis, mild nonspecific lymphocytic inflammation (yellow circles), and reactive epithelial changes. The erosive changes were more prominent in the distal esophagus. Original magnification, ×200 for all panels.

Additional infectious and autoimmune evaluation was unrevealing. Serologic and molecular tests were negative for HSV types 1 and 2, varicella-zoster virus, CMV, Candida albicans, and Mycoplasma pneumoniae. Immunobullous testing was negative, including serum anti-BP180 and anti-BP230 IgG antibodies, anti-desmoglein 1 and anti-desmoglein 3 IgG antibodies, and indirect immunofluorescence for IgG and IgA antibodies against the basement membrane zone.

She was started on viscous lidocaine for odynophagia, and her diet was gradually advanced. Upon discontinuation of the medication, the patient’s symptoms slowly began to improve. The primary recommendation was strict avoidance of further oxcarbazepine exposure without the need for immunosuppressive or additional pharmacologic therapy. At two-month follow-up, her oral ulceration, cheilitis, and odynophagia had resolved; however, she continued to report rare, intermittent dysphagia to solid foods. Videofluoroscopic swallowing assessment demonstrated intact oromotor function and normal transit of a 13-mm barium tablet through the upper esophageal sphincter. The Robust Esophageal Swallowing Test demonstrated appropriate esophageal clearance of liquids and pills but prolonged retention of a solid bolus in the proximal and mid-esophagus, consistent with mild esophageal phase dysphagia.

Given intermittent solid-food dysphagia, a repeat EGD was performed five months after the initial hospitalization. By this time, the patient reported continued improvement in her swallowing, with only rare dysphagia. EGD demonstrated complete normalization of the esophageal mucosa, with resolution of the previously described endoscopic abnormalities (Figure 2). Empiric esophageal dilation was performed with Savary dilators up to 51 Fr without resistance, mucosal tear, or bleeding. Esophageal biopsies showed benign squamous mucosa with focal vascular congestion, without eosinophilic or lymphocytic infiltration, dysplasia, or viral cytopathic effect (Figure 4). Repeat DIF was negative for IgG, IgM, IgA, and C3 deposition. The clinical course, including medication exposure, symptom progression, diagnostic evaluation, and outcome, is summarized in Table 2.

**Figure 4:**
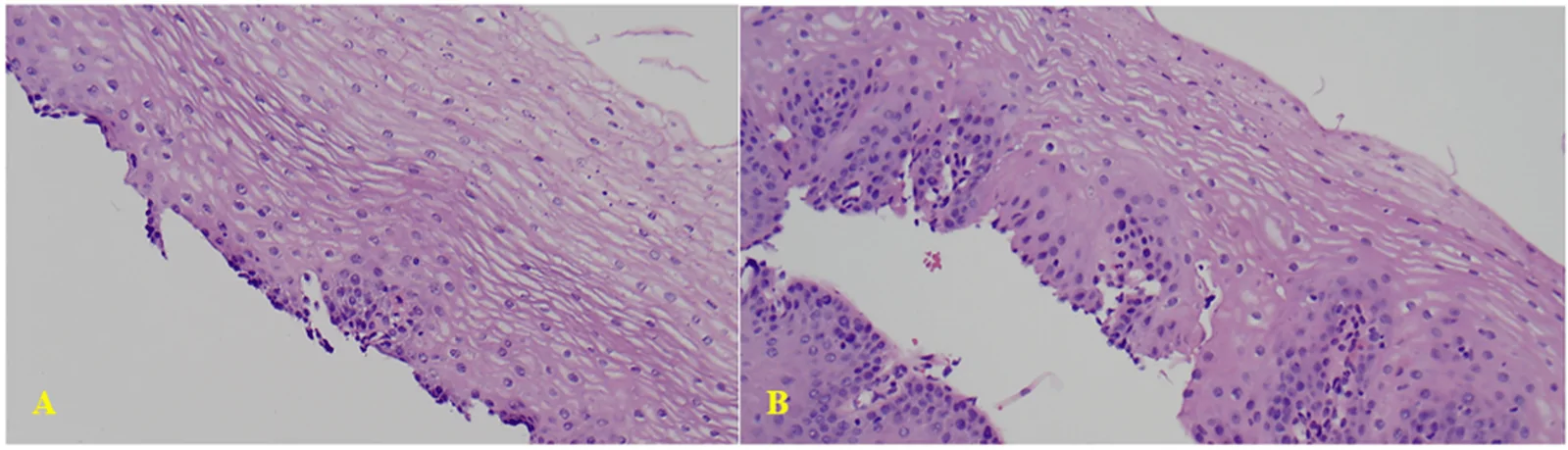
Histopathologic findings from esophageal biopsies obtained during follow-up upper endoscopy. Hematoxylin and eosin-stained sections obtained five months after discontinuation of oxcarbazepine demonstrate normal esophageal squamous mucosa without evidence of inflammation or mucosal injury (A, B). Original magnification, ×200 for both panels.

**Table 2:** Clinical timeline. EGD, esophagogastroduodenoscopy; HSV, herpes simplex virus

Time point	Event
Week 0	Oxcarbazepine 150 mg twice daily initiated for vestibular paroxysmia.
Week 2	Oxcarbazepine dose escalated to 300 mg twice daily per otolaryngology instructions.
Week 2, Day 1	Lip pruritus developed within 24 hours of dose escalation.
Week 2, Days 2-5	Progressive oral ulceration, lip edema, and tongue dysesthesia. Outside dermatology started empiric HSV infection treatment without improvement.
Week 3	Patient experienced worsening odynophagia and dysphagia. She was transferred to our institution, and oxcarbazepine was discontinued on the day of transfer.
Admission	Index EGD showed diffuse esophageal mucosal erythema, congestion, luminal narrowing, and multiple overlapping esophageal webs.
Discharge	Symptomatic improvement with Magic Mouthwash and gradual diet advancement.
Two-month follow-up	Resolution of oral ulceration, cheilitis, and odynophagia; the patient had persistent intermittent solid food dysphagia.
Five-month follow-up	Follow-up EGD showed complete endoscopic and histologic normalization.

## Discussion

Immune-mediated adverse drug reactions represent a diagnostically challenging spectrum of conditions characterized by unpredictable onset, variable clinical phenotype, and varying extent of organ involvement, with no universal classification system to guide phenotypic attribution [1]. Aromatic anticonvulsants, including oxcarbazepine, are among the most frequently implicated drug classes, with a well-established association with severe hypersensitivity syndromes, including SJS, TEN, and DRESS, historically described as anticonvulsant hypersensitivity syndrome (AHS) [1,3-5]. Although esophagitis is listed as an adverse event in the oxcarbazepine prescribing information, no published case report has described its clinical presentation, endoscopic findings, or histopathologic features [6]. Several lines of evidence suggest that oxcarbazepine was the causative agent in this patient, including the temporal relationship between dose escalation and symptom onset, systematic exclusion of alternative etiologies, and complete endoscopic and histologic resolution following drug withdrawal. Application of the Naranjo Adverse Drug Reaction Probability Scale yielded a score of seven (Table 3), consistent with a probable adverse drug reaction [7]. This case characterizes isolated oroesophageal mucosal injury with predominantly lymphocytic inflammation as a rare manifestation of an oxcarbazepine adverse drug reaction.

**Table 3:** Naranjo Adverse Drug Reaction Probability Scale assessment of oxcarbazepine-associated esophagitis.

Criterion	Score applied	Application to this case
1. Are there previous conclusive reports on this reaction?	0	No prior published case of oxcarbazepine-induced esophagitis identified.
2. Did the adverse event appear after the suspected drug was given?	+2	Yes
3. Did the adverse reaction improve when the drug was discontinued?	+1	Yes
4. Did the adverse reaction reappear when the drug was re-administered?	0	Not performed
5. Are there alternative causes that could have caused the reaction?	+2	No, infectious, eosinophilic, immunobullous, lichenoid, and cutaneous etiologies were excluded.
6. Did the reaction reappear when a placebo was given?	0	Not applicable
7. Was the drug detected in blood or other fluids in toxic concentrations?	0	Not applicable
8. Was the reaction more severe when the dose was increased?	+1	Yes, symptoms worsened after escalation from 150 mg to 300 mg twice daily.
9. Did the patient have a similar reaction to the same or similar drugs previously?	0	No prior exposure to oxcarbazepine or related aromatic anticonvulsants reported.
10. Was the adverse event confirmed by any objective evidence?	+1	Yes
Total Naranjo score	7	Probable adverse drug reaction (score 5-8)

Beyond the temporal association, several clinical and endoscopic features favored an immune-mediated drug reaction rather than direct mucosal toxicity. Pill esophagitis typically produces focal ulceration at areas of physiologic esophageal narrowing, most commonly in the mid-esophagus, with otherwise normal surrounding mucosa [2,8]. In contrast, our patient demonstrated diffuse circumferential mucosal congestion, sloughing, and multiple esophageal webs involving nearly the entire esophagus, with distal predominance. Widespread esophageal involvement of this severity and distal predominance cannot be explained by localized contact injury at a single point of pill transit. Furthermore, the concomitant oral mucosal involvement and approximately two-week latency between drug initiation, dose escalation, and symptom onset are more consistent with an immune-mediated hypersensitivity reaction than with direct mucosal toxicity.

Establishing oxcarbazepine as the causative agent further required a systematic exclusion of infectious, autoimmune, and dermatologic etiologies that can produce similar clinical and endoscopic presentations. Infectious esophagitis was excluded by negative polymerase chain reaction testing for HSV types 1 and 2, varicella-zoster virus, and Candida albicans from oral swabs. Esophageal biopsies were additionally negative for fungal elements on Grocott methenamine silver staining and negative for CMV and HSV on immunohistochemistry [9]. Eosinophilic esophagitis was excluded on the basis that current diagnostic criteria require a peak eosinophil count of at least 15 per high-power field, which was absent on targeted biopsies from both endoscopic examinations [10]. In addition, eosinophilic esophagitis does not produce the oral mucosal findings seen in this patient. Immunobullous disorders were excluded by negative serum BP180 and BP230 antibodies, negative desmoglein 1 and desmoglein 3 antibodies, and negative DIF for IgG, IgM, IgA, and C3 deposition on esophageal biopsies at both endoscopies [11-12]. Esophageal lichen planus was also considered given the mucosal sloughing and oral mucosal ulcers [13]; however, negative DIF findings and complete clinical, endoscopic, and histologic resolution following oxcarbazepine discontinuation argued against a chronic mucosal inflammatory disorder. SJS and TEN were excluded by the absence of cutaneous rash, skin tenderness, and a negative Nikolsky sign, as both are fundamentally diseases of the skin with secondary mucosal involvement [14]. DRESS was excluded by the absence of peripheral eosinophilia, fever, systemic rash, and systemic organ involvement [15]. Reactive infectious mucocutaneous eruption was less likely given the absence of preceding respiratory illness, fever, or conjunctival involvement and the close temporal relationship between symptom onset and oxcarbazepine dose escalation [16].

Prior reports of anticonvulsant-associated esophageal injury have occurred exclusively within systemic hypersensitivity syndromes. Balatsinou et al. described two cases of carbamazepine-induced eosinophilic esophagitis as a manifestation of AHS, while Greenish et al. reported esophageal eosinophilic infiltration associated with carbamazepine-induced DRESS [17-18]. Given the structural similarity between carbamazepine and oxcarbazepine, these reports provide biologic plausibility for a shared hypersensitivity mechanism among aromatic anticonvulsants causing esophageal injury. However, unlike those cases, our patient developed isolated oroesophageal mucosal injury without rash, eosinophilia, or multiorgan involvement, and biopsies demonstrated lymphocytic rather than eosinophilic inflammation. This distinction broadens the clinical spectrum of anticonvulsant-associated esophageal injury and suggests that localized mucosal hypersensitivity reactions may occur independently of the classic DRESS phenotype [19].

Diagnosis of immune-mediated adverse drug reactions remains dependent on clinical pattern recognition, causality assessment, and supportive histologic and laboratory findings, in the absence of a universal classification framework for atypical presentations with isolated organ involvement [1,19]. Additional cases are needed to better define the endoscopic phenotype, histologic pattern, and clinical course of oroesophageal mucosal injury as a rare adverse effect of oxcarbazepine. Further reporting may clarify whether lymphocytic mucosal involvement is specific to oxcarbazepine or represents a shared adverse effect among structurally related aromatic anticonvulsants.

## Conclusions

This case expands the recognized spectrum of oxcarbazepine-associated hypersensitivity reactions by demonstrating severe, reversible esophageal injury with concomitant oral mucosal involvement in the absence of eosinophilia, cutaneous, or systemic organ involvement. The temporal relationship to drug exposure, exclusion of alternative etiologies, and complete clinical, endoscopic, and histologic resolution following drug withdrawal suggest that oxcarbazepine is the most likely trigger. Even severe drug-induced esophageal injury may follow a favorable clinical course when the offending agent is recognized and withdrawn promptly. Clinicians should therefore consider medication-induced hypersensitivity reactions in the differential diagnosis of diffuse esophagitis and mucositis, as early identification may prevent unnecessary diagnostic testing and therapeutic interventions while facilitating complete recovery.
